# A comprehensive analysis of Y-chromosomal diversity in Colombian populations

**DOI:** 10.1007/s00414-026-03848-4

**Published:** 2026-05-28

**Authors:** Julyana Ribeiro, Zehra Köksal, Adriana Castillo, Adriana Ibarra, Beatriz Martinez, Humberto Ossa, Masinda Nguidi, Juan David Granda, Verónica Gomes, Pedro Rodrigues, Maria Inês Machado, Vania Pereira, Leonor Gusmão

**Affiliations:** 1 DNA Diagnostic Laboratory (LDD), Universidade do Estado do Rio de Janeiro, Rio de Janeiro, Brazil; 2 Section of Forensic Genetics, Department of Forensic Medicine, Faculty of Health and Medical Sciences, University of Copenhagen, Copenhagen, Denmark; 3 Department of Basic Sciences, Universidad Industrial de Santander (UIS), Bucaramanga, Colombia; 4 Institute of Biology, School of Natural and Exact Sciences (FCEN), IdentiGEN – Genetic Identification Laboratory and Research Group of Genetic Identification, University of Antioquia, Medellin, Antioquia Colombia; 5 Institute for Immunological Research, University of Cartagena, Cartagena, Colombia; 6 Facultad de Ciencias, Pontificia Universidad Javeriana, Bogotá, Colombia; 7 Laboratorio de Genética y Biología Molecular, Bogotá, Colombia; 8 Instituto de Investigação e Inovação em Saúde (i3S), Porto, Portugal; 9 Institute of Molecular Pathology and Immunology, University of Porto (IPATIMUP), Porto, Portugal; 10 Faculty of Sciences, University of Porto (FCUP), Porto, Portugal

**Keywords:** Y chromosome, Ancestry, Admixed population, Population substructure, Paternal ancestry, PowerPlex Y23

## Abstract

An intense gene flow between Native, European, and African groups, since the colonial period, has led to complex admixture patterns throughout Colombia. To investigate this genetic structure, we analyzed 23 Y-STRs in 975 males from five major regions of the country: Andes, Pacific, Caribbean, Orinoquía, and Amazon. To explore the paternal lineages and to reconstruct the underlying historical and demographic processes, a subset of 175 individuals was genotyped for 859 Y-SNPs using massively parallel sequencing. A high haplotypic diversity was found for the 23 Y-STRs (0.9998), compared to other Latin American populations studied. Pairwise genetic distance analyses (*F*_ST_ and *R*_ST_) showed a clearer differentiation among Colombian regions, when increasing the number of Y-STRs from 17 to 23. In population comparisons, Caribbean and Orinoquía regions clustered closer to European and Admixed populations, while the Pacific region moves towards African populations. The Amazon clustered with Peru and Ecuador that have high Native American ancestry. The predominant paternal ancestry in the Caribbean and Andes was European (79% and 87.9%, respectively), with macrohaplogroup R being the most frequent (39% and 64%, respectively). In the Caribbean region, sub-Saharan African lineages are the second most prevalent (16%), while in the Andean region the Native American lineages are the second most represented (7.6%), followed by a smaller proportion of sub-Saharan African lineages (4.5%). The results attest to the extensive regional genetic diversity within the country. The Y-STRs showed good intra and inter-population discrimination capabilities and support the establishment of specific regional databases for forensic purposes.

**Supplementary Information:**

The online version contains supplementary material available at 10.1007/s00414-026-03848-4.

## Introduction

Due to its haploid nature and advantageous location within the non-recombining region, Y-chromosomal specific markers are commonly applied in forensic investigations and population genetic research [1]. The forensic application of this type of markers depends on the availability of haplotypic data that allows an accurate estimation of the weight of evidence. Nonetheless, haplotype data for Y-chromosomal STRs (Y-STRs) included in the kits currently used in forensics are still scarce for Colombian populations.

Colombia is located in northwestern South America, sharing borders with Ecuador and Peru to the south, and Venezuela and Brazil to the east. It faces the Pacific Ocean to the west and the Caribbean Sea to the north. The country comprises 32 administrative departments and a Federal District, and it is geographically divided into six natural regions: Caribbean, Andes, Orinoquía, Pacific, Amazon, and Insular.

Colombia exhibits high ethnic diversity, primarily resulted from extensive admixture between the Native American populations and people arriving in the territory during the Spanish colonization that started in the 16th century. During this period, the Native American people faced enslavement, displacement, or extermination. Subsequently, during the transatlantic slave trade, Africans arrived in the Colombian territory, mainly through the port of Cartagena. Initially, enslaved African people that were taken to Cartagena came mainly from Upper Guinea [2]. In the mid-17th century, Angola became the main source of enslaved individuals, a situation that lasted until 1640, when the Spanish lost access to Portuguese trade entrepôts for human trafficking [2]. After that, the enslaved Africans arriving in Cartagena were brought mainly from the Gold Coast and Bight of Benin regions, until the beginning of the 18th century [2]. In the last century, migratory movements were mainly from neighboring countries [3].

In 2024, the Colombian population was approximately 53 million. According to the latest census conducted by the Colombian Administrative Department of Statistics in 2018 [4], the Colombian population is largely concentrated in urban areas, with 77.1% living in municipal capitals and 15.8% are in rural zones. The population is mostly concentrated in the Andean region, followed by the Caribbean. The forested regions of Colombia, comprising the Orinoquía and Amazon, are the largest in land area but remain the least densely populated. The indigenous population, self-declared within 115 distinct ethnic groups, corresponds to 4.4% of the total population [4]. They are distributed across 24 departments, mainly covering rural areas, with smaller proportions in urban centers. The indigenous population is prevalent in the eastern departments of the Orinoquía and Amazon regions, accounting for more than 57% of the population. In the province of La Guajira, in the Caribbean region, the indigenous population is also significant, corresponding to approximately 48% of the population. Afro-descendant groups represent 9.34% of the total Colombian population, and are concentrated in the Caribbean and Pacific Coasts, as well as in the Insular region.

As for most South American populations, a high genetic diversity has been reported in Colombia. Studies using ancestry-informative autosomal markers (AIMs) show distinct admixture patterns across the country. In admixed populations, the European component generally predominates, except in the Amazon and Pacific regions, where the Native American and African components are, respectively, higher [5].The Native American component is the second most prevalent throughout the country, except in the Caribbean region, where the African component is higher than the Native American one [5–8]. Contrasting results have been reported when analyzing the non-recombining mtDNA and Y-chromosomal genomes in admixed populations. A predominance of maternal lineages from Native American ancestry have been reported in populations from the Andean, Caribbean and Pacific regions [9–11]. For the paternal lineages, European ancestry prevails in most populations, except for those on the Insular region [9, 10, 12–16].

The main aim of this study was to expand Y-STR haplotype data for forensic use in admixed Colombian populations. A single study was identified that report Colombian population data [17] for the currently used forensic Y-STR kits, namely the Yfiler Plus (Thermo Fisher Scientific) and the PowerPlexY23 (Promega Corporation). Most studies included a subset of the loci in current forensic kits [9, 12, 18–28], and only a few analyzed all the 17 loci present in the Yfiler kit (Thermo Fisher Scientifc) [14, 16, 29, 30]. Moreover, for the Yfiler Plus and PowerPlex 23 kits, no Colombian data are currently available at the Y-Chromosome Haplotype Reference Database (YHRD, Release R69; https://yhrd.org). Data included in the YHRD comprise shorter datasets, namely 1,507 Y17 profiles, 1,857 Y12 profiles, and 3,453 profiles for the Minimal haplotype (Release R69).

In this study, a sample of 975 unrelated admixed individuals from the five main regions of Colombia (Amazon, Andean, Caribbean, Orinoquía, and Pacific) was selected and genotyped for the 23 Y-STRs included in the PowerPlex 23 kit (PPY23). This allowed the dataset available for forensic and population studies in Colombia to be considerably expanded, both in terms of the geographic scope of previous studies and the number of loci analyzed. Particularly, (i) samples from the Orinoquía were included, a region that had only been previously analyzed for 6 Y-STRs [9]; (ii) data for the Pacific and Insular regions was only available for a set of 17 Y-STRs (*n* = 58 [17], and *n* = 54 [14], respectively); (iii) Yfiler Plus data is only available for Andes, Caribbean and Amazon (*n* = 699 [16, 17, 30], *n* = 353 [17, 29], *n* = 65 [17], respectively); and (iv) no data were previously available for the full set of Y-STRs from the PowerPlexY23.

Given the distinct admixture processes from various continental origins that contributed to the present-day Colombian population, differences are expected in the genetic composition of populations from different regions of the country. Population substructure affects accuracy of forensic parameters and should be considered in haplotype databases. Therefore, to evaluate male-mediated admixture patterns, a subset of 175 samples from this study were also genotyped for 859 Y-SNPs, using the Ion AmpliSeq™ HID Y-SNP Research Panel v1 [31].

## Materials and methods

### Population sampling

**Fig. 1:**
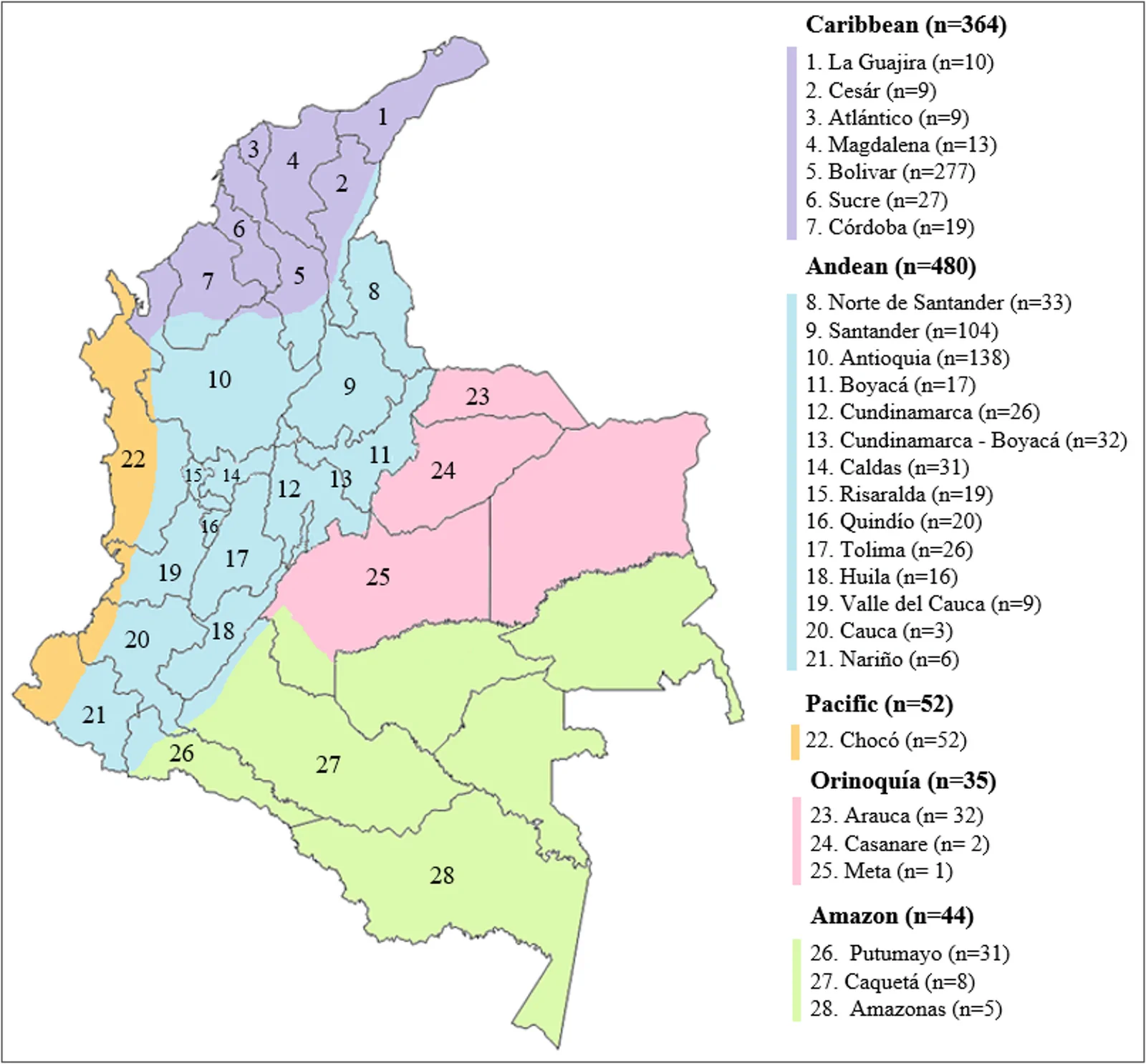
Map of Colombia showing sampling locations and the number of samples collected by natural region and Department

A total of 975 unrelated males from 27 Colombian departments along the five main natural regions of the country were selected for this study (see sampling locations and sizes in Fig. 1). DNA extraction was performed from blood samples using a Chelex method [32] or a salting-out protocol as described by Miller et al. [33].

This research follows the ethical principles stated in the Helsinki Declaration (2000) of the World Medical Association. The samples were collected under written informed consent. The project and informed consents were approved by the ethics committees from the Universidad Industrial de Santander (CEINCI − 11/08/2023) and from the University of Cartagena (approval number: 040).

## Y-STR genotyping

All samples were genotyped for 23 Y-STRs using the PowerPlex Y23 kit (PPY23), following the manufacturer’s recommendations (Promega Corporation, Madison, WI). Amplified fragments were separated and detected on a 3500 Genetic Analyzer and genotyped using the GeneMapper™ Software v4.1 (Applied Biosystems).

## Y-SNP genotyping

A total of 175 samples were randomly selected from Andes (*n* = 66), Caribbean (*n* = 100) and Amazon (*n* = 9) regions. This subset is representative of the Andean and Caribbean regions, where more than 90% of the population is concentrated [4], with differences expected compared to other regions, based on Y-STR results. Samples were genotyped for Y-SNPs using the Ion AmpliSeq™ HID Y-SNP Research Panel v1 [31] developed for massively parallel sequencing in the IonS5 System (Thermo Fisher Scientific). The panel contains 884 Y-SNPs located in 602 amplicons with an average amplicon size of 130 bp [31].

DNA amplification was performed by combining 4 µL of Ion AmpliSeq™ HiFi Mix, 10 µL of Ion AmpliSeq™ primer pool (2X), 1–5 µL of gDNA template (1-5ng) and the remaining volume of water to complete the 20 µL PCR reaction. A negative control was used in each PCR batch to confirm the absence of contamination. Library preparation was performed according to the manufacturer’s protocol.

Eluted libraries were quantified using the TaqMan™ Library Quantitation Kit (Thermo Fisher Scientific) and pooled to a final concentration of 50–80 pM. Libraries with concentrations below 80 pM were re-amplified with 8 cycles according to the manufacturer’s manual (Precision ID Library Kit on the Ion Torrent system by Thermo Fisher Scientific Pub. No. MAN0015802) to guarantee enough genomic material for normalization.

Template preparation and chip loading were performed on the Ion Chef™ Instrument (Thermo Fisher Scientific). 32 libraries were loaded into each Ion 530™ Chip (Thermo Fisher Scientific). Sequencing was performed on the Ion PGM S5™ instrument (Thermo Fisher Scientific) with 650 run flows, using Ion S5™ Sequencing Kit (Thermo Fisher Scientific).

### Statistical analysis

Data analyses were performed after grouping data from this study with published data from Colombia. From the available studies, we have selected those from admixed populations (excluding Native or Afro-descent communities) with complete haplotype profiles for the Y-Filer STRs (Supplementary Table S1) [14, 16, 17, 29, 30].

The NEVGEN software (https://www.nevgen.org/) was used to predict haplogroups from PPY23 haplotypes in samples with intermediate, duplicated, or null alleles. In cases where NEVGEN attributed a probability of less than 90%, the haplogroup assignment was confirmed using previously described SNaPshot multiplexes [34].

For Y-SNP data, coverage statistics for the 602 target regions were generated using the Coverage Analysis plugin v5.10.0.3. A minimum coverage threshold of 20 reads per amplicon was applied for quality trimming. The Y-leaf software version 3.0 [35] was run for Y-SNP variant calling and to infer the haplogroups using BAM files. The threshold criteria for the haplogroup inference were set as follows: minimum of 20 reads for each base, quality threshold of 20 for each base read, and majority base threshold of 95%. The Integrative genomics viewer (IGV) software was used to inspect ambiguous variation [36].

Haplogroup frequencies were calculated by direct counting. The haplotype/haplogroup diversities (HD) and the total number of observed and unique haplotypes were calculated using the Arlequin software v3.5.1.2 [37]. The same software was used in population differentiation analyses of Colombian samples, based on pairwise genetic distances (*F*_ST_ and *R*_ST_), and in the analysis of molecular variance (AMOVA). In multiple tests, the significance level of 0.05 was adjusted by sequential Bonferroni procedure [38].

In pairwise genetic distance analyses, the number of repeats in DYS389I was subtracted in DYS389II. Moreover, for *R*_ST_ analyses DYS385 was excluded, and microvariant alleles, duplications, and null alleles were coded as “?”.

Pairwise genetic distances (*F*_ST_ and *R*_ST_) based on 23 Y-STR haplotypes were also calculated between our population samples and populations from Europe and Africa, as well as Native American and other admixed populations from South America (Supplementary Table S1) [39–44].

Multi-Dimensional Scaling (MDS) plots of pairwise genetic distances were generated with the software STATISTICA v.14.0.0.15 (TIBCO Software Inc.).

A Y-STR-based phylogenetic network was built for R-V88 chromosomes from African and European populations [45–53] for haplotypes defined by 8 loci shared with the available datasets and using the software Network 4.6.1.1 (Fluxus-Engineering), with the median-joining algorithm.

## Results and discussion

### Haplotype diversity and allelic variation

Haplotype profiles and haplogroups obtained in this study are shown in Supplementary Table S2.

A high value of HD (0.9998 ± 0.0001) was observed in the general sample from Colombia, consistent with those reported in other admixed South American populations [39–41, 43, 54, 55]. In the Colombian samples analyzed, 913 distinct haplotypes were identified: 861 were singletons (observed only once), 44 were shared by two individuals, six by three individuals, and two haplotypes were shared by four individuals.

An allele 6 at DYS391 locus was detected in four Colombian samples. Although rare, this allele is represented in YHRD in samples from East Asia, and with a lower frequency in admixed populations from South America. It was previously reported in the Andean region of Colombia and associated with a novel Native American lineage within Q1a2-M346*(xM3) [16]. In this study, the allele DYS391*6 was also detected in the Andes, as well as in both the Caribbean and Amazon regions in samples predicted to belong to haplogroup Q1b1a2-Z780, according to results from the NEVGEN online tool (three samples with probabilities ≥ 98.1% and one sample with a probability of 42.2%).

Intermediate, duplicated and null alleles were detected in 64 out of the 975 unrelated male samples genotyped in this study (Table 1). To investigate the origin of these alleles, haplogroups of four samples were attributed based on Y-SNP genotyping using the Ion AmpliSeq™ HID Y-SNP Research Panel v1. For the other 60 samples, haplogroups were predicted from each corresponding haplotype using the NEVGEN software. In 20 samples, haplogroups were predicted with less than 92% probability. Of these, 16 samples were genotyped for relevant Y-SNPs by SNaPshot, and the results were compatible with the haplogroup predicted by NEVGEN in all cases. No reliable results were obtained for four samples from the Andes, namely (i) one carrying a DYS458*17.2 allele, assigned to J1a2a1a2-P58; (ii) one carrying a DYS458*15,16 duplication, with no haplogroup predicted (100% Unsupported subclade); (iii) one carrying a DYS448*19,20 duplication, assigned to E1a-M132; and (iv) one carrying a DYS 458*16.2 allele, assigned to J1a2a1a2-P58.

Seventeen different intermediate alleles were observed in the complete dataset at the following loci: DYS437, DYS458, DYS385, DYS570, DYS635, and DYS438 (Table 1). Intermediate alleles at Y-STRs have been widely reported at various loci in different populations. In the present dataset, DYS458 exhibited the highest number of intermediate alleles, followed by DYS385. For both markers, intermediate alleles were detected in samples from different regions, with no geographic specificity being found (Table 1).

**Table 1:** Intermediate, duplicated and null alleles detected in a population sample of 975 unrelated males from Colombia

Y-STR	Genotype	Region (*n*)	NEVGEN - Haplogroup Predicted^#^
Intermediate alleles			
DYS385	17,17.2	Caribbean (2)	A1a-M31
DYS385	10.2,12	Andean (1)	R1b-DF27
DYS385	11,13.2	Andean (1) / Caribbean (1)	*R1b1a1a2a1a2a1b1a / R1b-U152
DYS385	11.2,12	Andean (1)	R1b-DF27
DYS385	13,14.2	Caribbean (1)	J2a1-L26
DYS385	13.2,16	Caribbean (1)	R1b-V88
DYS437	13.2	Andean (1)	R1b-DF27
DYS437	14.2	Andean (1)	R1b-U106
DYS438/DYS458	9.2/19.2	Andean (3)	J1a3-Z1828
DYS458	16.2	Andean (3) / Caribbean (3)	1 R1b-Z2103 / 5 J1a2a1a2-P58
DYS458	17.2	Andean (6) / Caribbean (6) / Orinoquía (1)	12 J1a2a1a2-P58 / 1 *J1a2a1a2d2b2b2c
DYS458	18.2	Andean (5) / Caribbean (1) / Pacific (1)	6 J1a2a1a2-P58 / 1 *J1a2a1a2d2b2b2c4c
DYS458	19.2	Andean (1) / Caribbean (2) / Pacific (1)	3 J1a3-Z1828 / 1 J1a-PH77
DYS458	20.1	Caribbean (2)	1 E1b1b-M81 /1 J1a-PH77
DYS458	20.2	Caribbean (1)	J1a3-Z1828
DYS570	17.2	Andean (1)	R1b-DF27
DYS635	23.1	Andean (1)	R1b-DF27
Duplications			
DYS389I/DYS437	12,13/14,15	Caribbean (1)	R1b-L51
DYS448	18,20	Andean (1)	R1b-U106
DYS448	20,21	Caribbean (2)	E1a M132
DYS448	19,20	Andean (1) / Caribbean (1) / Orinoquía (1)	1 R1b-P312 / 2 E1a-M132
DYS448	19,21	Caribbean (1)	E1a-M132
DYS456	15,16	Andean (1)	100% Unsupported subclade
DYS576	15,17	Pacific (2)	E1b1a-V38
Null alleles			
DYS19/DYS392	0	Andean (1)	R1b-L21
DYS390	0	Andean (1)	R1b-L21
DYS448	0	Andean (3) / Caribbean (1)	3 R1a-M198 / 1 *R1b1a1a2a1a1c2b2a1b1b1a

^#^Haplogroups predicted by the NEVGEN with probability below 90% were confirmed by genotyping diagnostic Y-SNPs by SNaPshot, with no discrepancies observed. *Haplogroup assigned based on Y-SNP genotyping using the Ion AmpliSeq™ HID Y-SNP Research Panel v1

Samples with DYS385 intermediate alleles were assigned to haplogroups A1a, J2a1, and different sub-lineages within R1b, indicating that these intermediate alleles likely arose from multiple independent events. It is noteworthy that the two haplotypes classified within haplogroup A1a, in addition to allele 17.2 in DYS385 also had allele 6 at DYS533, a rare variant found just in one sample in the YHRD Admixed Metapopulation. Most samples with non-consensus alleles at DYS458, including three individuals simultaneously carrying 9.2 at DYS438 and 19.2 at DYS458, were classified within J1a, except one that was assigned to the R1b haplogroup. This finding aligns with previous associations of DYS458 intermediate alleles with J1a-M267 and R1b3-M405 haplogroups [56]. The remaining intermediate alleles at DYS437, DYS570 and DYS635 were assigned to sub-lineages within haplogroup R1b, which was the most frequent in the studied Colombian samples. The low number of observations does not allow inferences regarding the origin of these alleles.

Duplicated alleles were observed at DYS448, DYS456, DYS576, DYS389I and DYS437 (Table 1). Deletions, duplications, and triplications are frequently observed in ampliconic regions of the Y chromosome, since this chromosome is under low selective pressure [57]. On the long arm of the Y chromosome, the Azoospermia Factor regions (AZFa, AZFb, AZFc) are susceptible to frequent structural rearrangements. Y-STRs used in forensic genetics that are located inside these regions (e.g. DYF387S1, DYS385, DYS389I/II, DYS391, DYS392, DYS437, DYS438, DYS439, DYS448, DYS460, DYS549 and DYS635) tend to present duplications and/or deletions, as a result of non-allelic homologous recombination between palindromes [58–61]. This mechanism can explain the duplications involving both DYS437 and DYS389I (Table 1), located in the AZFa [62], as well as the seven duplications at DYS448, located in the AZFc. The lack of association of the DYS448 duplications with a specific haplogroup point to at least three independent duplication events. Balaresque et al. [58] identified DYS448 duplications in samples belonging to haplogroups O3e, C*, C3c, G, O2, D*, and E3b, supporting independent deletion and duplication events within AZF regions, making it challenging to assign a specific geographic origin to the duplications and null alleles observed in this study.

DYS456 and DYS576 are both located on the short arm of the Y chromosome. For the sample with the duplication at DYS456, the NEVGEN software could not predict any haplogroup. The two samples with duplication at DYS576 are from the Pacific region, and they were predicted as belonging to the African haplogroup E1b1a. This finding is consistent with the previous detection of this duplication in a sample from Benin [39] and with the high levels of African ancestry in the Colombian Pacific region [5].

Null alleles were also observed at DYS448, DYS390, DYS19 and DYS392. In the case of DYS448, the null alleles can also be due to non-allelic homologous recombination within the AZFc, which explains the 4 instances observed in Andes and Caribbean within two different haplogroups (R1a and R1b).

Null alleles at DYS390, DYS19 and DYS392 are outside palindromic regions and, therefore, they most likely resulted from mutations at the primer binding regions. Null alleles at these loci were described in Ghana [44] and South Africa [63, 64], and reported in YHRD in admixed South American, Native American and African populations. In our samples, the haplotype carrying null alleles simultaneously at DYS19 and DYS392, as well as the haplotype with a null allele at DYS390, were predicted to belong to haplogroup R1b-L21.

## Comparison of Y-STR haplotypes in Colombian populations

**Fig. 2:**
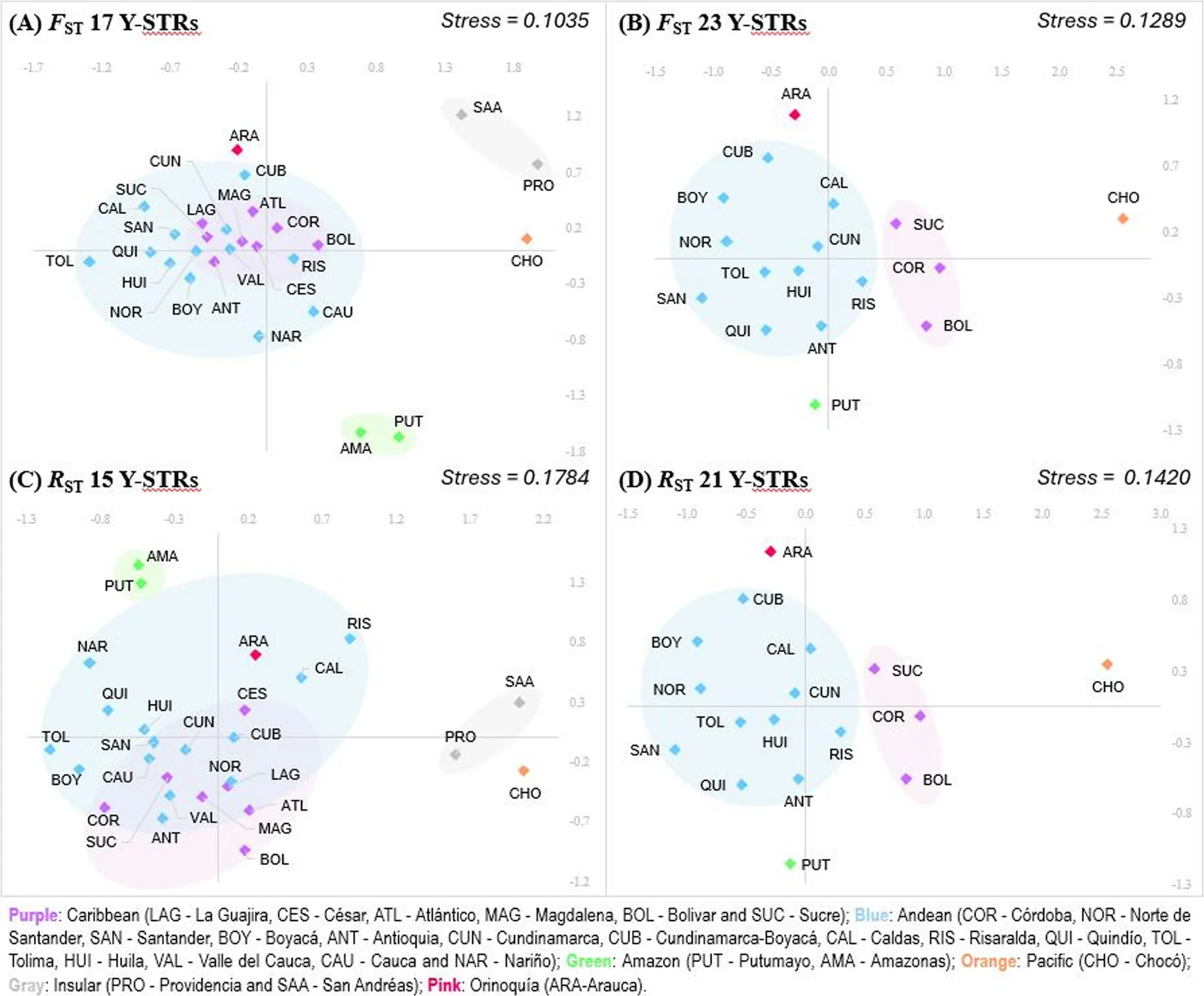
MDS plots based on *F*_ST_ and *R*_ST_ genetic distances. The number of loci varied in each analysis. In the *F*_ST_ analysis the complete sets of 17 Y-STRs (**A**) and 23 Y-STRs (**B**) were included, while in the *R*_ST_ analysis only 15 Y-STRs (**C**) and 21 Y-STRs (**D**) were used, by excluding DYS385 loci. Purple, gray and blue circles include population samples from the same Colombian region with non-statistically significant differentiation and genetic distances below 2%

To assess population structure across Colombian departments and regions, pairwise genetic distances (*F*_ST_ and *R*_ST_) were calculated using data from this study and from previous publications that included at least the Y17 loci (Supplementary Tables S1 and S3). Departments with less than 16 haplotypes were excluded from these analyses: Casanare (*n* = 2), Meta (*n* = 1) and Caquetá (*n* = 6). Genetic distances were calculated for both 17 Y-STRs and 23 Y-STRs, with less populations available for the larger Y-STR set (Supplementary Table S1). The results obtained are presented in Supplementary Table S3 and represented in an MDS plot (Fig. 2).

In all analyses, Putumayo and Amazonas showed no significant differences among them, and appeared genetically distinct from all other departments, showing significant differences with all departments except Risaralda. This pattern is probably related to the higher Native American ancestry of the Amazonian populations when compared to other regions, which is supported by data from ancestry-informative markers [5].

A significant genetic differentiation from other departments was also evident for the department of Chocó, which only showed non-significant low differences when compared with Providencia and San Andrés for the 15 Y-STR set, probably due to shared African ancestry [8]. Studies using ancestry-informative markers also identified statistically significant differences between Andean populations (Antioquia, Bogotá, Norte de Santander) and Chocó [5, 65].

Among the Andean populations, significant differentiation was obtained for the department of Cauca, Nariño and Tolima in both *F*_ST_ and *R*_ST_ analyses for the smaller Y-STR set (Fig. 2A and C). It is worth noting that Nariño and Cauca showed the lowest genetic distances values with Putumayo, which is geographically close. The geographic proximity of these departments to the Amazon may have contributed to gene flow and, possibly, a greater Native American ancestry than the other departments in the Andean region. Indeed, a previous study using ancestry-informative markers showed that the southwestern Andean region, which comprises the departments of Nariño, Cauca, and part of Putumayo, preserved a significant proportion of Native American ancestry (37–44%) [5].

When comparing the five geographic regions of Colombia, the populations from Orinoquía, Andes, and Caribbean tend to cluster together (Fig. 2), with an overlap of the clusters of the Andes and Caribbean populations in the analyses performed for a smaller set of markers (Fig. 2A and C). In the analyses performed with 21 and 23 Y-STRs, a clearer separation of the five regions is observed (Fig. 2B and D).

Taken as a whole, the genetic distance results obtained demonstrate differences in paternal lineages between the five geographic regions of Colombia, which should be considered when evaluating forensic evidence. Although some differences were also observed between populations within the regions, the overall results did not reveal a statistically significant differentiation.

To assess the robustness of the groupings based on the genetic distances between pair of populations defined in Fig. 2, an AMOVA analysis was performed. Colombian departments included in the present study, together with previously published data [14, 16, 17, 29, 30], were grouped into six major regions: Insular, Andes, Caribbean, Orinoquía, Pacific, and Amazon for the analysis of 17-STRs and 23-STRs. The results obtained from the two sets of Y-STR markers indicated that majority of genetic variation occurred within populations (> 97.81%), while up to 1.53% of the variation was attributed to differences among groups, and less than 0.66% to differences among populations within groups.

Although the genetic differentiation between Colombian departments was low in both sets, it was statistically significant (*F*_ST_ > 0.01914 and *p* = 0.00000). Also, values of among groups’ fixation index (*F*_CT_) were low (< 0.05), indicating small but statistically significant genetic differences among the regional groupings.

In the AMOVA based on 23 Y-STRs, values of among populations within groups’ fixation index (*F*_SC_) were not statistically significant (*F*_SC_ = 0.00179, *p* = 0.16109). On the other hand, with the 17-STRs marker set, significant genetic differences were detected among populations within the regions (*F*_SC_ = 0.00737, *p* = 0.00000). These findings suggest that an increase in the number of Y-STR markers appears to improve the resolution for identifying regional differences. Thus, based on the AMOVA results and genetic distance analyses, the regional groupings of the Colombian departments established for the 23 Y-STR markers set were adopted for further analyses.

Finally, it is worth noting that sampling in the Andean and Caribbean regions far exceeds that of Amazon, Pacific, and Orinoquía regions, reducing the statistical power and precision of estimates involving these regions. Therefore, future studies should increase sampling efforts in genetically unique but sparsely populated regions to achieve balanced sample sizes across regions, enabling more robust inter regional comparisons.

## Comparisons of admixed South American populations

**Fig. 3:**
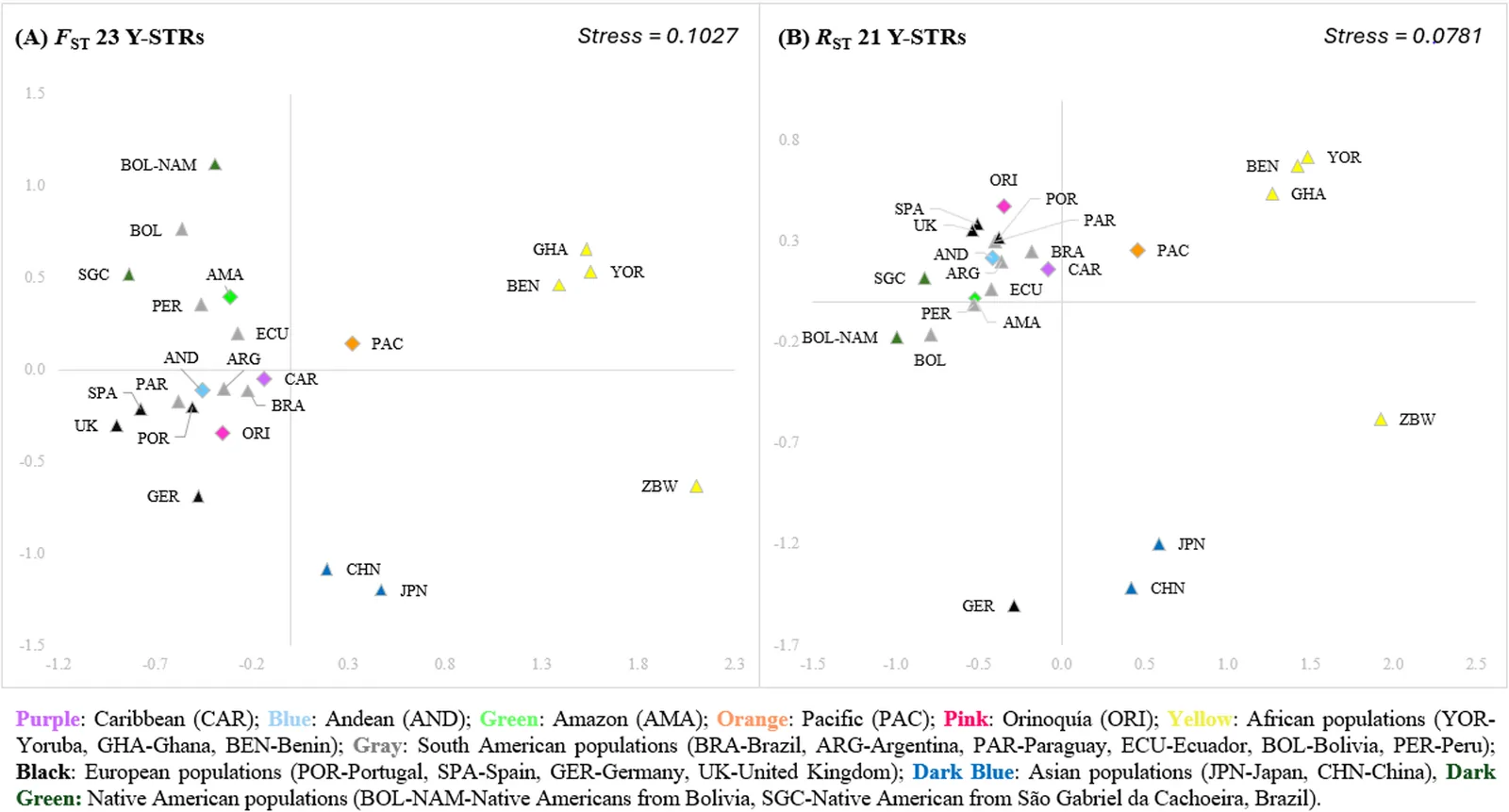
MDS plot of populations comparisons. A) MDS plot based on *F*_ST_ genetic distances between Colombian regions and other populations, based on 23 Y-STRs (B) MDS plot based on *R*_ST_ genetic distances between Colombian regions and other populations, based on 21 Y-STRs, by excluding DYS385 loci

For comparative purposes, PPY23 haplotype datasets from the Colombian regions were compared with those from admixed South American, Native American, European, East Asian and African populations, for which data were available for the same set of markers. The results are presented in Supplementary Table S4. As shown in Fig. 3A, populations from the Andean, Caribbean, and Orinoquía regions cluster with European and other admixed populations, indicating stronger genetic affinities with Argentina, Paraguay and Brazil, all showing high genetic proximity to Iberian populations [41, 43, 60]. These results are historically consistent, as the colonization of South America was predominantly carried out by settlers from the Iberian Peninsula beginning in the early 15th century. It is also important to highlight that Germany appears more distant from this cluster, particularly according to the *R*_ST_ results.

Although pairwise genetic distances (*F*_ST_ and *R*_ST_) indicate statistically significant differentiation between Colombian regions and African groups, the Pacific region still shows a greater proximity to the western African populations. As previously reported for Y-SNPs in the Caribbean in this study, this region exhibits a significantly higher frequency of sub-Saharan lineages, highlighting stronger genetic affinities with African populations.

The Amazon population clusters with Peru and Ecuador, showing no significant differences, likely due to the strong Native American component shared among these groups. However, although the Amazonian population is genetically closer to Bolivia, the increased distance and significant differences relative to Bolivian natives suggest that the Andean Native component of Bolivia is distinct from the Amazonian component found in Colombia.

The Amazon population clusters with Peru and Ecuador, showing no significant differences, likely due to the strong Native American component shared among these groups. However, although the Amazonian population is genetically closer to Bolivia, the increased distance and significant differences relative to Bolivian natives suggest that the Andean Native component of Bolivia is distinct from the Amazonian component found in Colombia.

### Y-SNP analysis and haplogroup diversity in the Andes and Caribbean regions

#### Haplogroup frequencies and diversity

In agreement with what was previously reported [66], seven of the 602 amplicons failed to meet the 20x threshold in ≥ 90% of the samples and were excluded from downstream analyses. Despite this, haplogroup assignment was still possible since the panel contained alternative Y-SNPs for the same haplogroup, or downstream Y-SNPs relative to those that failed.

From the 859 Y-SNPs claimed to be included in the panel, two of the Y-SNPs are in regions outside the amplified amplicons, 16 Y-SNPs were excluded for lower coverage, and 569 Y-SNPs were not variable in the samples analyzed, presenting the ancestral allele in all samples. The final dataset consisted of 272 variable Y-SNPs (i.e., with ancestral and derived states in the 175 samples), defining 64 haplogroups.

Yleaf prediction results for the 175 samples are provided in Supplementary Table S2. Five Andean samples presented an additional derived allele that has been observed within the context of other haplogroup phylogenies. One sample classified as I-M36 (AN475 in Supplementary Figure S1) presented a derived allele at M7 usually observed within haplogroup O. Two samples from haplogroup Q-M3 (AN437 and AN444), one from haplogroup R-V88 (AN471), and one from haplogroup J-L283 (AN424) presented a derived allele in variant L729, originally reported within haplogroup N [31].

These positions were all above the coverage threshold, but the majority base threshold was below 95% for three of them. Further inspection on the IGV software confirmed that these positions were not sequencing artefacts and may represent recurrent mutations (Supplementary Figure S1). Indeed, in the ISOGG Tree (https://isogg.org/tree/index.html) L729 is associated to sub-lineages inside haplogroups R (L729.2) and J (L729.2), in the UYSD (https://ysnp.erasmusmc.nl/ysnp_database/) and YFull (https://www.yfull.com/tree/) it is described within haplogroup N, and Yleaf included this mutation as diagnostic for haplogroup R1b1a1b1a1a1b1a1a. To the best of our knowledge, position M7 has not been previously reported as recurrent, and L729 has not been observed in haplogroup Q or R-V88 backgrounds. More studies will be needed to understand if these positions define novel branches within haplogroups I2a1a1, Q1b1a1a and R1b1a2.

**Fig. 4:**
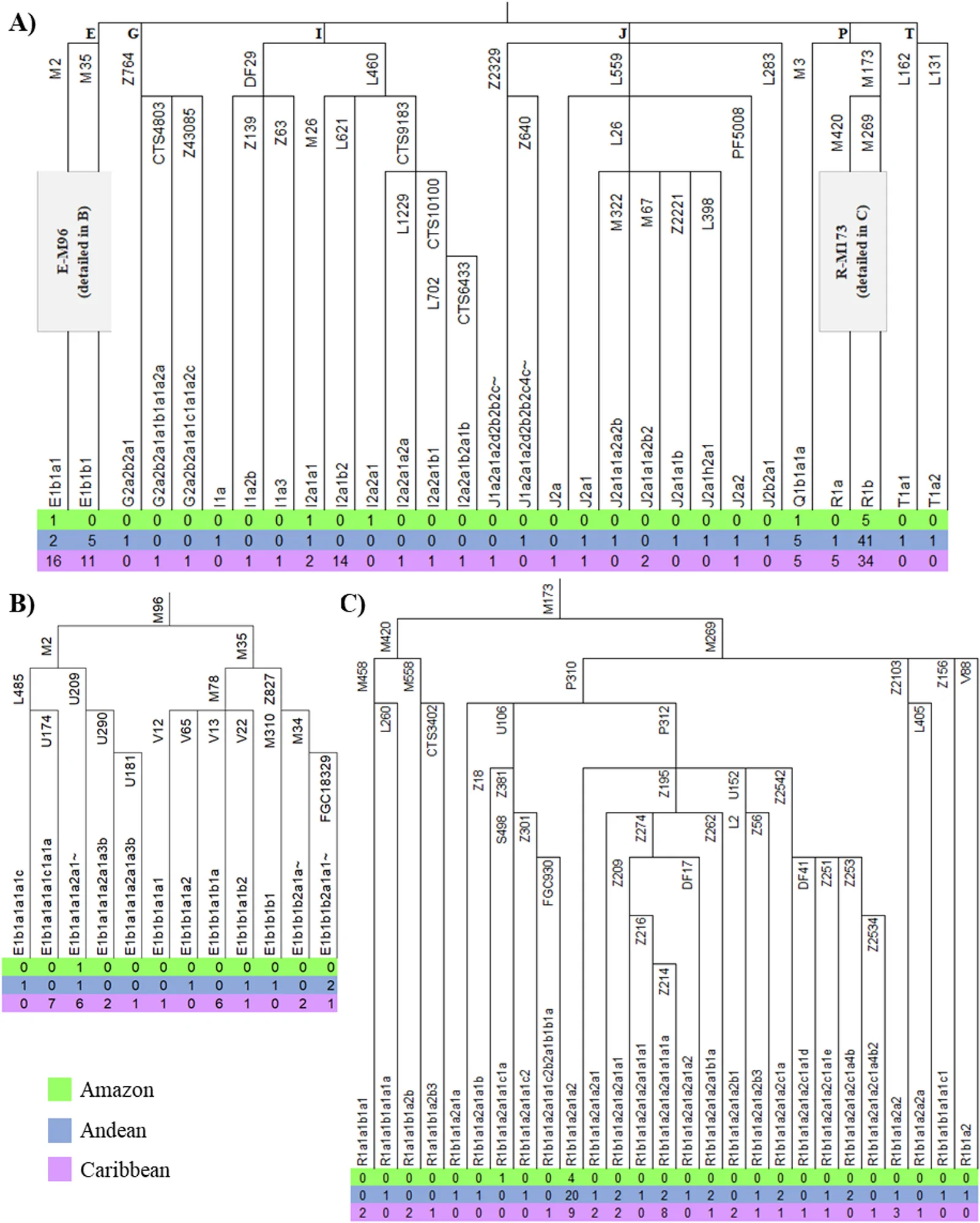
Phylogenetic tree of Y-haplogroups analyzed and their absolute frequencies in Colombian regions

The frequencies of the haplogroups detected in the Andean, Caribbean and Amazon regions are shown in Fig. 4A. Since only nine Y chromosomes were analyzed from the Amazon region, these samples were not used to make population inferences.

The macrohaplogroup R-M173 was the most prominent haplogroup in both Colombian regions, with frequencies of 64% in the Andean region and 39% in the Caribbean. This lineage is subdivided in R1a-M420, found in Eastern Europe, and R1b-M269, more frequent in Western and Central European populations [67–69]. Lineages inside the R1b-P312 branch predominate in both the Caribbean and the Andes, with a frequency of 85% within haplogroup R1b-M269 (Fig. 4B). This is one of the most frequent haplogroups in Western Europe and particularly in the Iberian Peninsula [67, 70]. A high diversity of R1b-P312 sub-lineages were found in our data.

A sample from haplogroup R-V88 was observed in our Colombian dataset. This lineage is more frequent in Africa [46, 71], although it has been also described in current populations from Sardinia and Corsica islands [52, 53, 71]. To determine the most likely origin of this R-V88 lineage, a network was constructed using haplotypes with 8 Y-STRs from Sub-Saharan African and European populations (Supplementary Figure S2). The Colombian haplotype shows greater affinity with African haplotypes and, therefore, its continental origin was inferred to be African.

Other European lineages were also identified in Colombia. The G sub-lineages represented 1.81% of our dataset, while haplogroup I represented 13.86%. Sub-haplogroup I showed the highest incidence in Caribbean. Haplogroup J was identified in 7.83% of the Colombia samples, and haplogroup T was the less frequent (1.2%). Although haplogroups G, I, J and T can be found in other continents [72–77], in the historical context of Caribbean and Andean populations, these lineages were most likely introduced by Europeans.

Haplogroup E was more frequent in the Caribbean (27%) than in Andean region (10.6%). In the Caribbean, the most frequent sub-haplogroup was E-M2 (Fig. 4C), widely distributed in sub-Saharan Africa and associated with the Bantu expansion [46, 78]. The presence of E-M2 lineages in Colombia most likely resulted from transatlantic slave trade, mainly from West and Central-West Africa, as well as from Mozambique on the East African coast. On the other hand, Andes exhibited a higher frequency of lineages inside sub-haplogroup E-M35 (Fig. 4C). E-M35 lineages (E-M78; E-M310 and E-M34) are frequent both in Eurasia and Africa [74, 79, 80].

Concerning clade Q, only the Q-M3* lineage was detected in the present study with a low frequency in the total dataset (6.02%). Haplogroup Q is found approximately in 85% of Native Americans [75, 81, 82]. The low frequencies observed in both the Caribbean and Andes are consistent with historical records documenting low Y-chromosomal Native ancestry, due to sex-biased admixture between Native women and non-Native men, mostly from Europe [9].

The high diversity of lineages found in our samples reflects the intense migratory flow and admixture resulting from the arrival of colonizers from the Iberian Peninsula, accompanied by enslaved Africans. Future studies with a broader analysis of the distribution of Y-STR haplogroups, including the Pacific, Amazonian, and Orinoco regions, would be relevant for a more comprehensive view of Colombian history, particularly for African lineages linked to the transatlantic slave trade and for Native American lineages associated with early migrations.

### Colombian ancestry

Lineages belonging to haplogroups E-M35, G, I, J, R-M173*(xV88) and T are consistent with a predominant European paternal contribution in Colombia and were inferred to have been introduced during colonization and later reinforced by more recent European migrations into the country. Although a Middle Eastern contribution of lineages from haplogroups E, G, J and T cannot be fully excluded, considering the historical and migratory movements to Colombia, the introduction of these lineages was most likely mediated by males coming from Europe. An African ancestry was attributed to the remaining E-lineages, all of them carrying the M2 derived allele, as well as to haplogroup R-V88 detected in one sample (Fig. 4). Native American ancestry was inferred by the proportion of samples belonging to haplogroup Q-M3, and no sub-lineages were detected inside this haplogroup.

The largest proportion of paternal lineages in Andean and Caribbean regions was of European origin, 87.9% and 79.0%, respectively. In the Andes, Native American ancestry (7.6%) exceeded African ancestry (4.5%), whereas in the Caribbean, African lineages (16.0%) were more frequent than the Native American ones (5.0%).

These findings are in accordance with previous studies showing that European lineages represent the main contributor of the paternal ancestry in Colombia due to the Spanish colonization of this country [16]. The higher frequency of sub-Saharan African lineages in the Caribbean region, also previously reported by others [15], can be explained by the role of Cartagena as one of the most important ports in South America during the transatlantic slave trade.

### Forensic relevant parameters

Molecular diversity parameters were recalculated using the complete haplotype dataset for Colombia, considering the regions and the department’s populations. High levels of haplotype diversity (HD ≥ 99.92%; Table 2) were observed for all regions of Colombia, except for Orinoquía. The Andean region was the only region that shared haplotypes with the others (six haplotypes with the Caribbean, one with Orinoquía, and three with Amazon). In the studied populations, the HD values were similar to those previously reported from Colombia admixed individuals analyzed with YFiler [14, 16, 17, 29, 30]. Urban populations such as those analyzed in this study, are expected to show high levels of admixture and uniparental-lineage diversity due to colonization, the slave trade, and more recent immigration waves [5, 9, 16].

**Table 2:** Diversity for 23 Y-STR haplotypes and Y-SNP haplogroups for Colombian regions

Population	Y-STR haplotypes			Y-SNP haplogroups		
	*N*	Different haplotypes	Haplotype diversity	*N*	Different haplogroups	Haplogroup diversity
Andes	480	454	0.9997 ± 0.0002	66	38	0.9068 ± 0.0323
Caribbean	364	343	0.9997 ± 0.0002	10	41	0.9539 ± 0.0093
Orinoquía	35	29	0.9832 ± 0.0131	-	-	-
Pacific	52	51	0.9992 ± 0.0040	-	-	-
Amazon	44	44	1.0000 ± 0.0048	9	6	0.8333 ± 0.1265
Total sample	975	913	0.9998 ± 0.0001	-	-	-

Given that different marker sets commonly used in forensics include Y-STRs with differential mutation rates, the probability of finding at least one haplotype difference between related individuals due to mutation depends on the average mutation rate and the number of analyzed loci. In Table 3 are the proportion of related males (separated by 1 to 5 meiosis) that are expected to present different haplotypes, considering previously reported mutation rates [83–85]. The results show that the average mutation rate among loci is higher for PPY23 compared to previous kits including fewer markers, but lower than for larger kits. The Yfiler Plus marker set shows the highest average mutation rate, reflecting the inclusion of rapid mutating markers. As expected, the probability of differentiation between related individuals increases with the inclusion of a greater number of markers.

**Table 3:** List of Y-STR kits with corresponding number of markers (N), average mutation rate over markers, and the expected probability of finding at least one haplotype difference between individuals separated by 1 to 5 meiosis

Marker set	*N*	Av. mutation rate	Expected frequency of different haplotypes between individuals separated by one to five meiosis*				
			1	2	3	4	5
Minimal	8	0.22%	1.76%	3.48%	5.18%	6.84%	8.48%
PPY12	11	0.22%	2.41%	4.77%	7.07%	9.31%	11.50%
YFiler	16	0.27%	4.18%	8.18%	12.01%	15.69%	19.21%
PPY23	22	0.34%	7.29%	14.05%	20.32%	26.13%	31.52%
Yfiler Plus and STRtyper-27	25	0.49%	11.64%	21.93%	31.02%	39.06%	46.15%
Argus-28	27	0.47%	12.02%	22.60%	31.91%	40.10%	47.30%
AGCU Y37	34	0.45%	14.30%	26.56%	37.06%	46.07%	53.78%
Yfiler Platinum	35	0.44%	14.44%	26.79%	37.36%	46.41%	54.15%
PathFinder Plus and GoldenEye	37	0.43%	14.66%	27.17%	37.85%	46.96%	54.74%

*Calculated according to Palha et al. [86]

## Conclusions

This study provides a comprehensive overview of paternal lineages and their regional patterns of variation within Colombia. The analysis of Colombian haplotypes in this study reinforces the extensive regional genetic diversity within the country. Genetic distance results among regions revealed marked differences in paternal lineages across the five main geographical regions of Colombia, preventing the use of a unified national database of Y-haplotype frequencies for an accurate evaluation of forensic evidence at regional level. This diversity pattern, also reported in studies using other genetic markers, reflects the country’s colonization history, which begins with the exclusive presence of Native American groups, followed by European colonization in 15th century, and subsequently the forced introduction of enslaved Africans.

As a consequence, genetic distance analyses based on Y-STR data from different Colombian regions, compared with reference populations of European, African, and admixed South American groups, unsurprisingly indicate closer affinities with Iberian populations, particularly for Andean, Orinoquía, and Caribbean regions. This pattern is further supported by the predominance of the European haplogroup R1b-P312 among male lineages in the Andes and Caribbean, a lineage commonly found at high frequencies in the Iberian Peninsula, reinforcing historical records of the predominant contribution of Iberian settlers. In contrast, the Amazonian region shows closer affinities with Native American populations, whereas the Pacific region displays stronger genetic proximity to African groups.

The studied populations exhibited high HD, consistent with what has been observed for South American populations shaped by admixture from at least three different continental sources. The PowerPlex Y23 markers demonstrated a high capacity for intrapopulation discrimination among Colombian regional groups, supporting their use in forensic applications.

## Supplementary Information

Below is the link to the electronic supplementary material.

Supplementary Material 1

Supplementary Material 2

## References

[1] Kayser M (2017) Forensic use of Y-chromosome DNA: a general overview. Hum Genet 136(5):621–635. 10.1007/s00439-017-1776-9 28315050 10.1007/s00439-017-1776-9 PMC5418305

[2] Borucki A, Eltis D (2020) D. Wheat. From the Galleons to the Highlands: Slave Trade Routes in the Spanish Americas. University of New Mexico, New Mexico

[3] Departamento Administrativo Nacional de Estadística (DANE) (2022) Registro Estadístico de Migración Internacional – REMI. Reporte Estadístico de Migracion n°2. https://www.dane.gov.co/files/investigaciones/poblacion/migracion/2doreporte-migracion.pdf. Access 29 Jan 25

[4] Departamento Administrativo Nacional de Estadística (DANE) Censo Nacional de Población y Vivienda 2018. http://www.dane.gov.co/censo. Access 01 Dec 25

[5] Ossa H, Aquino J, Pereira R et al (2016) Outlining the Ancestry Landscape of Colombian Admixed Populations. PLoS ONE 11(10):e0164414. 10.1371/journal.pone.0164414 27736937 10.1371/journal.pone.0164414 PMC5063461

[6] Homburger JR, Moreno-Estrada A, Gignoux CR et al (2015) Genomic Insights into the Ancestry and Demographic History of South America. PLoS Genet 11(12):e1005602. 10.1371/journal.pgen.1005602 26636962 10.1371/journal.pgen.1005602 PMC4670080

[7] Carvalho Gontijo C, Porras-Hurtado L, Freire-Aradas A et al (2020) A population informative multiplex for the Americas. Forensic Sci Int Genet 44:102200. 10.1016/j.fsigen.2019.102200 31760353 10.1016/j.fsigen.2019.102200

[8] Mogollón Olivares F, Madero JM, Casas-Vargas A et al (2020) Contrasting the ancestry patterns of three distinct population groups from the northernmost region of South America. Am J Phys Anthropol 173(3):437–447. 10.1002/ajpa.24130 32856314 10.1002/ajpa.24130

[9] Rojas W, Parra MV, Campo O et al (2010) Genetic make up and structure of Colombian populations by means of uniparental and biparental DNA markers. Am J Phys Anthropol 143(1):13–20. 10.1002/ajpa.21270 20734436 10.1002/ajpa.21270

[10] Criollo-Rayo AA, Bohórquez M, Prieto R, CHIBCHA Consortium et al (2018) Native American gene continuity to the modern admixed population from the Colombian Andes: Implication for biomedical, population and forensic studies. Forensic Sci Int Genet 36:e1–e7. 10.1016/j.fsigen.2018.06.006 29909140 10.1016/j.fsigen.2018.06.006

[11] Castillo A, Rondón F, Mantilla G et al (2023) Maternal ancestry and lineages diversity of the Santander population from Colombia. Forensic Sci Res 8(3):241–248. 10.1093/fsr/owad032 38221971 10.1093/fsr/owad032 PMC10785602

[12] Carvajal-Carmona LG, Soto ID, Pineda N et al (2000) Strong Amerind/white sex bias and a possible Sephardic contribution among the founders of a population in Northwest Colombia. Am J Hum Genet 67(5):1287–1295. 10.1016/S0002-9297(07)62956-5 11032790 10.1016/s0002-9297(07)62956-5 PMC1288568

[13] Acosta MA, Blanco-Verea A, Lareu MV et al (2009) The genetic male component of two South-Western Colombian populations. Forensic Sci Int Genet 3(2):e59–61. 10.1016/j.fsigen.2008.06.006 19215870 10.1016/j.fsigen.2008.06.006

[14] Alonso LA, Usaquén W (2013) Y chromosome and surname analysis of the native islanders of San Andrés and Providencia (Colombia). Homo 64(1):71–84. 10.1016/j.jchb.2012.11.006 23290785 10.1016/j.jchb.2012.11.006

[15] Noguera MC, Schwegler A, Gomes V et al (2014) Colombia’s racial crucible: Y chromosome evidence from six admixed communities in the department of bolivar. Ann Hum Biol 41(5):453–459. 10.3109/03014460.2013.852244 24215508 10.3109/03014460.2013.852244

[16] Alonso Morales LA, Casas-Vargas A, Castro MR et al (2018) Paternal portrait of populations of the middle Magdalena River region (Tolima and Huila, Colombia): New insights on the peopling of Central America and northernmost South America. PLoS ONE 13(11):e0207130. 10.1371/journal.pone.0207130 30439976 10.1371/journal.pone.0207130 PMC6237345

[17] Casas-Vargas A, Guzmán AC, Avila AJ et al (2025) Genetic Structure of the Y-Chromosome in Colombia: An Analysis of Regional Diversity and Ancestry. Am J Hum Biol 37(12):e70171. 10.1002/ajhb.70171 41328020 10.1002/ajhb.70171

[18] Vidal-Martinez J, Benítez-Páez A (2003) Ossa. Population data of new Y-chromosome STRs GATA C4, DYS438, DYS437, GATA A7.2, GATA H4, DYS439 and GATA A10 in males from Colombia. Forensic Sci Int 135(3):243–246. 10.1016/s0379-0738(03)00204-4 12927406 10.1016/s0379-0738(03)00204-4

[19] Builes JJ, Bravo MLJ, Martínez-Pancorbo M et al (2004) Discrimination index of Y-chromosomal haplotypes in an Antioquia (Colombia) population sample. Int Congr Ser 1261:275–277. 10.1016/S0531-5131(03)01636-4

[20] Builes JJ, Bravo ML, Montoya A et al (2004) Population genetics of eight new Y-chromosomal STR haplotypes in three Colombian populations: Antioquia, Chocó and Cartagena. Int Congr Ser 1261:310–312. 10.1016/S0531-5131(03)01552-8

[21] Yunis JJ, Acevedo LE, Campo DS, Yunis EJ (2005) Population data of Y-STR minimal haplotypes in a sample of Caucasian-Mestizo and African descent individuals of Colombia. Forensic Sci Int 151(2–3):307–313. 10.1016/j.forsciint.2005.02.005 15939168 10.1016/j.forsciint.2005.02.005

[22] Gaviria AA, Ibarra AA, Palacio OD et al (2005) Y-chromosome haplotype analysis in Antioquia (Colombia). Forensic Sci Int 151(1):85–91. 10.1016/j.forsciint.2004.07.006 15935946 10.1016/j.forsciint.2004.07.006

[23] Builes JJ, Bravo ML, Gómez C et al (2006) Y-chromosome STRs in an Antioquian (Colombia) population sample. Forensic Sci Int 164(1):79–86. 10.1016/j.forsciint.2005.10.005 16289613 10.1016/j.forsciint.2005.10.005

[24] Builes JJ, Castañeda SP, Espinal C et al (2006) Analysis of 16 Y-chromosomal STRs in a Córdoba (Colombia) population sample. Int Congr Ser 1288:174–176. 10.1016/j.ics.2005.09.094

[25] Builes JJ, Martínez B, Gómez A et al (2007) Y chromosome STR haplotypes in the Caribbean city of Cartagena (Colombia). Forensic Sci Int 167(1):62–69. 10.1016/j.forsciint.2005.12.015 16455219 10.1016/j.forsciint.2005.12.015

[26] Yunis JJ, Acevedo LE, Campo DS, Yunis EJ (2013) Geno-geographic origin of Y-specific STR haplotypes in a sample of Caucasian-Mestizo and African-descent male individuals from Colombia. Biomedica 33(3):459–467. 10.7705/biomedica.v33i3.807 24652182 10.7705/biomedica.v33i3.807

[27] Núñez C, Baeta M, Ibarbia N et al (2017) 17 to 23: A novel complementary mini Y-STR panel to extend the Y-STR databases from 17 to 23 markers for forensic purposes. Electrophoresis 38(7):1016–1021. 10.1002/elps.201600313 27987217 10.1002/elps.201600313

[28] Baeta M, Núñez C, Villaescusa P et al (2018) Assessment of a subset of Slowly Mutating Y-STRs for forensic and evolutionary studies. Forensic Sci Int Genet 34:e7–e12. 10.1016/j.fsigen.2018.03.008 29588179 10.1016/j.fsigen.2018.03.008

[29] Romero RE, Briceño I, del Lizarazo R et al (2008) A Colombian Caribbean population study of 16 Y-chromosome STR loci. Forensic Sci Int Genet 2(2):e5–8. 10.1016/j.fsigen.2007.09.005 19083801 10.1016/j.fsigen.2007.09.005

[30] Avila SJ, Briceño I, Gómez A (2009) Genetic population analysis of 17 Y-chromosomal STRs in three states (Valle del Cauca, Cauca and Nariño) from Southwestern Colombia. J Forensic Leg Med 16(4):204–211. 10.1016/j.jflm.2008.12.002 19329077 10.1016/j.jflm.2008.12.002

[31] Ralf A, van Oven M, Montiel D, González et al (2019) Forensic Y-SNP analysis beyond SNaPshot: High resolution Y-chromosomal haplogrouping from low quality and quantity DNA using Ion AmpliSeq and targeted massively parallel sequencing. Forensic Sci Int Genet 41:93–106. 10.1016/j.fsigen.2019.04.001 31063905 10.1016/j.fsigen.2019.04.001

[32] Walsh PS, Metzger DA, Higuchi R (1991) Chelex 100 as a Medium for Simple Extraction of DNA for PCR-Based Typing from Forensic Material. Bio Techniques 10:506–513. 10.2144/000114018 1867860

[33] Miller SA, Dykes DD, Polesky HF (1988) A simple salting out procedure for extracting DNA from human nucleated cells. Nucleic Acids Res 16:1215. 10.1093/nar/16.3.1215 3344216 10.1093/nar/16.3.1215 PMC334765

[34] Simão F, Ribeiro J, Vullo C et al (2021) The Ancestry of Eastern Paraguay: A Typical South American Profile with a Unique Pattern of Admixture. Genes (Basel) 12(11):1788. 10.3390/genes12111788 34828394 10.3390/genes12111788 PMC8625094

[35] Ralf A, Montiel González D, Zhong K, Kayser M, Yleaf (2018) Yleaf: Software for Human Y-Chromosomal Haplogroup Inference from Next-Generation Sequencing Data. Mol Biol Evol. 2018, 35(5):1291-1294. 10.1093/molbev/msy032 10.1093/molbev/msy032 29518227

[36] Robinson JT, Thorvaldsdóttir H, Winckler W et al (2011) Integr Genomics Viewer Nat Biotechnol 29:24–26. 10.1038/nbt.1754 10.1038/nbt.1754 PMC3346182 21221095

[37] Excoffier L, Lischer HEL (2010) Arlequin suite ver 3.5: A new series of programs to perform population genetics analyses under Linux and Windows. Mol Ecol Resour 10:564–567. 10.1111/j.1755-0998.2010.02847.x 21565059 10.1111/j.1755-0998.2010.02847.x

[38] Benjamini Y (1995) Hochberg. Controlling the False Discovery Rate: A Practical and Powerful Approach to Multiple Testing. Royal Stat Soc J Ser B: Methodological 57:289–300. 10.1111/j.2517-6161.1995.tb02031.x

[39] Purps J, Siegert S, Willuweit S et al (2014) A global analysis of Y-chromosomal haplotype diversity for 23 STR *loci*. Forensic Sci Int Genet 12(100):12–23. 10.1016/j.fsigen.2014.04.008 24854874 10.1016/j.fsigen.2014.04.008 PMC4127773

[40] Toscanini U, Gaviria A, Pardo-Seco J et al (2018) The geographic mosaic of Ecuadorian Y-chromosome ancestry. Forensic Sci Int Genet 33:59–65. 10.1016/j.fsigen.2017.11.011 29197245 10.1016/j.fsigen.2017.11.011

[41] Ribeiro J, Romero M, Simão F et al (2018) Analysis of 23 Y-STRs in a population sample from eastern Paraguay. Forensic Sci Int Genet 37:e20–e22. 10.1016/j.fsigen.2018.08.007 30131298 10.1016/j.fsigen.2018.08.007

[42] Caputo M, Sala A, Corach D (2019) Demand for larger Y-STR reference databases in ethnic melting-pot countries: Argentina as a test case. Int J Legal Med 133:1309–1320. 10.1007/s00414-019-02012-5 30737602 10.1007/s00414-019-02012-5

[43] Ambrosio IB, Braganholi DF, Orlando LBM et al (2020) Mutational data and population profiling of 23 Y-STRs in three Brazilian populations. Forensic Sci Int Genet 48:102348. 10.1016/j.fsigen.2020.102348 32707472 10.1016/j.fsigen.2020.102348

[44] Kofi AE, Hakim HM, Khan HO et al (2020) Population data of 23 Y chromosome STR loci for the five major human subpopulations of Ghana. Int J Legal Med 134(4):1313–1315. 10.1007/s00414-019-02099-w 31154498 10.1007/s00414-019-02099-w

[45] Di Cristofaro J, Mazières S, Tous A et al (2018) Prehistoric migrations through the Mediterranean basin shaped Corsican Y-chromosome diversity. PLoS ONE 13(8):e0200641. 10.1371/journal.pone.0200641 30067762 10.1371/journal.pone.0200641 PMC6070208

[46] Berniell-Lee G, Calafell F, Bosch E et al (2009) Genetic and demographic implications of the Bantu expansion: insights from human paternal lineages. Mol Biol Evol 26(7):1581–1589. 10.1093/molbev/msp069 19369595 10.1093/molbev/msp069

[47] González M, Gomes V, López-Parra AM et al (2013) The genetic landscape of Equatorial Guinea and the origin and migration routes of the Y chromosome haplogroup R V88. Eur J Hum Genet: EJHG 21(3):324–331. 10.1038/ejhg.2012.167 22892526 10.1038/ejhg.2012.167 PMC3573200

[48] Fortes-Lima C, Brucato N, Croze M et al (2015) Genetic population study of Y-chromosome markers in Benin and Ivory Coast ethnic groups. Forensic Sci Int Genet 19:232–237. 10.1016/j.fsigen.2015.07.021 26275614 10.1016/j.fsigen.2015.07.021

[49] Larmuseau MHD, Vessi A, Jobling MA et al (2015) The paternal landscape along the Bight of Benin–Testing regional representativeness of West-African population samples using Y-chromosomal markers. PLoS ONE 10(11):e0141510. 10.1371/journal.pone.0141510 26544036 10.1371/journal.pone.0141510 PMC4636292

[50] Rosa A, Ornelas C, Jobling MA et al (2007) Y-chromosomal diversity in the population of Guinea-Bissau: a multiethnic perspective. BMC Evol Biol 7:124. 10.1186/1471-2148-7-124 17662131 10.1186/1471-2148-7-124 PMC1976131

[51] de Filippo C, Barbieri C, Whitten M et al (2011) Y-chromosomal variation in sub-Saharan Africa: insights into the history of Niger-Congo groups. Mol Biol Evol 28(3):1255–1269. 10.1093/molbev/msq312 21109585 10.1093/molbev/msq312 PMC3561512

[52] Morelli L, Contu D, Santoni F et al (2010) A comparison of Y-chromosome variation in Sardinia and Anatolia is more consistent with cultural rather than demic diffusion of agriculture. PLoS ONE 5(4):e10419. 10.1371/journal.pone.0010419 20454687 10.1371/journal.pone.0010419 PMC2861676

[53] Contu D, Morelli L, Santoni F et al (2008) Y-chromosome based evidence for pre-neolithic origin of the genetically homogeneous but diverse Sardinian population: inference for association scans. PLoS ONE 3(1):e1430. 10.1371/journal.pone.0001430 18183308 10.1371/journal.pone.0001430 PMC2174525

[54] Mut P, Bertoni B, Sapiro R et al (2023) Insights into the Y chromosome human diversity in Uruguay. Am J Hum Biol 35(12):e23963. 10.1002/ajhb.23963 37493343 10.1002/ajhb.23963

[55] Monte SMC, Sampaio B, Torres JCN et al (2025) Genetic characterization of paternal lineages by Y-STR in three sample populations in Northeastern Brazil. Int J Legal Med 139(2):541–549. 10.1007/s00414-025-03407-3 39775878 10.1007/s00414-025-03407-3

[56] Myres NM, Ekins JE, Lin AA et al (2007) Y-chromosome short tandem repeat DYS458.2 non-consensus alleles occur independently in both binary haplogroups J1-M267 and R1b3-M405. Croat Med J 48(4):450–459 17696299 PMC2080563

[57] Butler JM, Decker AE, Kline MC (2005) Vallone. Chromosomal Duplications Along the Y-Chromosome and Their Potential Impact on Y-STR Interpretation. J Forensic Sci 50(4):853–859. 10.1371/journal.pone.0137223 16078487

[58] Balaresque P, Bowden GR, Parkin EJ et al (2008) Dynamic nature of the proximal AZFc region of the human Y chromosome: multiple independent deletion and duplication events revealed by microsatellite analysis. Hum Mutat 29(10):1171–1180. 10.1002/humu.20757 18470947 10.1002/humu.20757 PMC2689608

[59] Turrina S, Caratti S, Ferrian M, De Leo D (2015) Deletion and duplication at DYS448 and DYS626 loci: unexpected patterns within the AZFc region of the Ychromosome. Int J Leg Med 129(3):449–455. 10.1007/s00414-015-1178-2 10.1007/s00414-015-1178-2 25821202

[60] Mayordomo AC, Gagliardi F, Simão F et al (2025) Using uniparental genetic profiles to unravel the complexity of Argentine admixed populations. Forensic Sci Int Genet 76:103216. 10.1016/j.fsigen.2024.103216 39732109 10.1016/j.fsigen.2024.103216

[61] Flores-Espinoza R, Nguidi M, Ribeiro J et al (2025) Deletion events at DYS448 locus: Exploring rearrangement patterns in the AZFc region of the Y chromosome. Proceedings of the 30th Congress of the International Society for Forensic Genetics. Universidade de Santiago de Compostela, pp. 1079–1084. 10.15304/cc.2025.1869

[62] Bosch E, Jobling M (2003) Duplications of the AZFa region of the human Y chromosome are mediated by homologous recombination between HERVs and are compatible with male fertility. Hum Mol Genet 12(3):341–347. 10.1093/hmg/ddg031 12554687 10.1093/hmg/ddg031

[63] Shabalala S, Ghai M, Okpeku M (2023) Analysis of Y-STR diversity and DNA methylation variation among Black and Indian males from KwaZulu-Natal, South Africa. Forensic Sci Int 348:111682. 10.1016/j.forsciint.2023.111682 37094501 10.1016/j.forsciint.2023.111682

[64] Dooley KB, Madisha MT, Strümpher S, Ehlers K (2022) Forensic genetic value of 27 Y-STR loci (Y-Filer^®^ Plus) in the South African population. Sci Justice 62(3):358–364. 10.1016/j.scijus.2022.03.009 35598928 10.1016/j.scijus.2022.03.009

[65] Pereira R, Alves C, Aler M et al (2018) A GHEP-ISFG collaborative study on the genetic variation of 38 autosomal indels for human identification in different continental populations. Forensic Sci Int Genet 32:18–25. 10.1016/j.fsigen.2017.09.012 29024923 10.1016/j.fsigen.2017.09.012

[66] Köksal Z, Burgos G, Carvalho E et al (2022) Testing the Ion AmpliSeq™ HID Y-SNP Research Panel v1 for performance and resolution in admixed South Americans of haplogroup Q. Forensic Sci Int Genetic 59:102708. 10.1016/j.fsigen.2022.102708 10.1016/j.fsigen.2022.102708 35453088

[67] Myres NM, Rootsi S, Lin AA et al (2011) A major Y-chromosome haplogroup R1b Holocene era founder effect in Central and Western Europe. Eur J Hum Genet 19(1):95–101. 10.1038/ejhg.2010.146 20736979 10.1038/ejhg.2010.146 PMC3039512

[68] Kayser M, Lao O, Anslinger K et al (2005) Significant genetic differentiation between Poland and Germany follows present day political borders, as revealed by Y-chromosome analysis. Hum Genet 117(5):428–443. 10.1007/s00439-005-1333-9 15959808 10.1007/s00439-005-1333-9

[69] Underhill PA, Poznik GD, Rootsi S et al (2015) The phylogenetic and geographic structure of Y-chromosome haplogroup R1a. Eur J Hum Genet: EJHG 23(1):124–131. 10.1038/ejhg.2014.50 24667786 10.1038/ejhg.2014.50 PMC4266736

[70] Valverde L, Illescas M, Villaescusa P et al (2016) New clues to the evolutionary history of the main European paternal lineage M269: dissection of the Y-SNP S116 in Atlantic Europe and Iberia. Eur J Hum Genet 24:437–441. 10.1038/ejhg.2015.114 26081640 10.1038/ejhg.2015.114 PMC4755366

[71] Cruciani F, Trombetta B, Sellitto D et al (2010) Human Y chromosome haplogroup R-V88: a paternal genetic record of early mid Holocene trans Saharan connections and the spread of Chadic languages. Eur J Hum Genet: EJHG 18(7):800–807. 10.1038/ejhg.2009.231 20051990 10.1038/ejhg.2009.231 PMC2987365

[72] Cinnioğlu C, King R, Kivisild T et al (2004) Excavating Y-chromosome haplotype strata in Anatolia. Hum Genet 114(2):127–148. 10.1007/s00439-003-1031-4 14586639 10.1007/s00439-003-1031-4

[73] Di Giacomo F, Luca F, Popa LO et al (2004) Y chromosomal haplogroup J as a signature of the post-neolithic colonization of Europe. Hum Genet 115(5):357–371. 10.1007/s00439-004-1168-9 15322918 10.1007/s00439-004-1168-9

[74] Semino O, Magri C, Benuzzi G et al (2004) Origin, diffusion, and differentiation of Y-chromosome haplogroups E and J: inferences on the neolithization of Europe and later migratory events in the Mediterranean area. Eur J Hum Genet 74(5):1023–1034. 10.1086/386295 10.1086/386295 PMC1181965 15069642

[75] Karafet TM, Mendez FL, Meilerman MB et al (2008) New binary polymorphisms reshape and increase resolution of the human Y chromosomal haplogroup tree. Genome Res 18(5):830–838. 10.1101/gr.7172008 18385274 10.1101/gr.7172008 PMC2336805

[76] Hammer MF, Behar DM, Karafet TM et al (2009) Extended Y chromosome haplotypes resolve multiple and unique lineages of the Jewish priesthood. Hum Genet 126(5):707–717. 10.1007/s00439-009-0727-5 19669163 10.1007/s00439-009-0727-5 PMC2771134

[77] Balanovsky O, Dibirova K, Dybo A et al (2011) Genographic Consortium. Parallel Evolution of Genes and Languages in the Caucasus Region. Mol Biol Evol 28(10):2905–2920. 10.1093/molbev/msr126 21571925 10.1093/molbev/msr126 PMC3355373

[78] Cruciani F, Santolamazza P, Shen P et al (2002) A back migration from Asia to sub-Saharan Africa is supported by high-resolution analysis of human Y-chromosome haplotypes. Eur J Hum Genet 70(5):1197–1214. 10.1086/340257 10.1086/340257 PMC447595 11910562

[79] Cruciani F, La Fratta R, Santolamazza P et al (2004) Phylogeographic analysis of haplogroup E3b (E-M215) y chromosomes reveals multiple migratory events within and out of Africa. Am J Hum Genet 74(5):1014–1022. 10.1086/386294 15042509 10.1086/386294 PMC1181964

[80] Cruciani F, Trombetta B, Novelletto A, Scozzari R (2008) Recurrent mutation in SNPs within Y chromosome E3b (E-M215) haplogroup: a rebuttal. Am J Hum Biol 20(5):614–616. 10.1002/ajhb.20790 18449920 10.1002/ajhb.20790

[81] Seielstad MT, Hebert JM, Lin AA et al (1994) Cavalli-Sforza. Construction of human Y-chromosomal haplotypes using a new polymorphic A to G transition. Hum Mol Genet 3(12):2159–2161. 10.1093/hmg/3.12.2159 7881413 10.1093/hmg/3.12.2159

[82] Jota MS, Lacerda DR, Sandoval JR et al (2016) Genographic Consortium. New native South American Y chromosome lineages. J Hum Genet 61(7):593–603. 10.1038/jhg.2016.26 27030145 10.1038/jhg.2016.26

[83] Antão-Sousa S, Gusmão L, Modesti NM et al (2024) Microsatellites’ mutation modeling through the analysis of the Y-chromosomal transmission: Results of a GHEP-ISFG collaborative study. Forensic Sci Int Genet 69:102999. 10.1016/j.fsigen.2023.102999 38181588 10.1016/j.fsigen.2023.102999

[84] Liu Z, Long G, Lang Y et al (2023) Sequence-based mutation patterns at 41 Y chromosomal STRs in 2 548 father-son pairs. Forensic Sci Res 8(2):152–162. 10.1093/fsr/owad016 37621447 10.1093/fsr/owad016 PMC10445670

[85] Ma X, Li R, Xie J et al (2026) A comprehensive evaluation of mutation rates and male differentiation using an 80-Y-STR panel. Forensic Sci Int Genet 83:103439. 10.1016/j.fsigen.2026.103439 41633138 10.1016/j.fsigen.2026.103439

[86] Palha T, Gusmão L, Ribeiro-Rodrigues E, Guerreiro JF, Ribeiro-Dos-Santos A, Santos S (2012) Disclosing the genetic structure of Brazil through analysis of male lineages with highly discriminating haplotypes. PLoS ONE 7(7):e40007. 10.1371/journal.pone.0040007 22808085 10.1371/journal.pone.0040007 PMC3393733

