# Supplementary material for: A comprehensive analysis of Y-chromosomal diversity in Colombian populations
Source: Int J Legal Med. 2026 May 28;140(5):2681–96. doi: 10.1007/s00414-026-03848-4 (PMC13499867; doi:10.1007/s00414-026-03848-4)
Supplement: Supplementary file 1 [file 414_2026_3848_MOESM1_ESM.pdf]

Supplementary Figures

A)

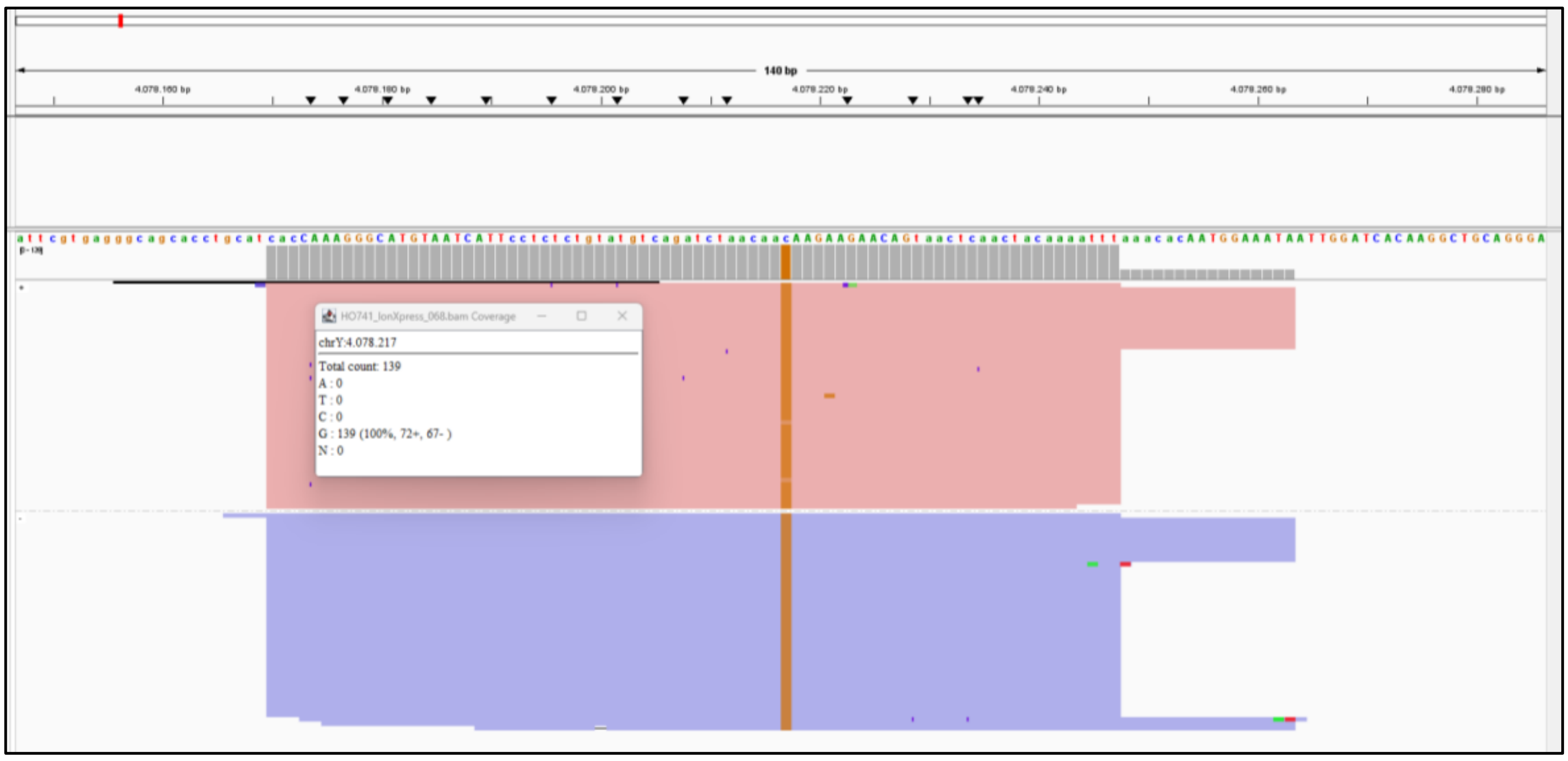

B)

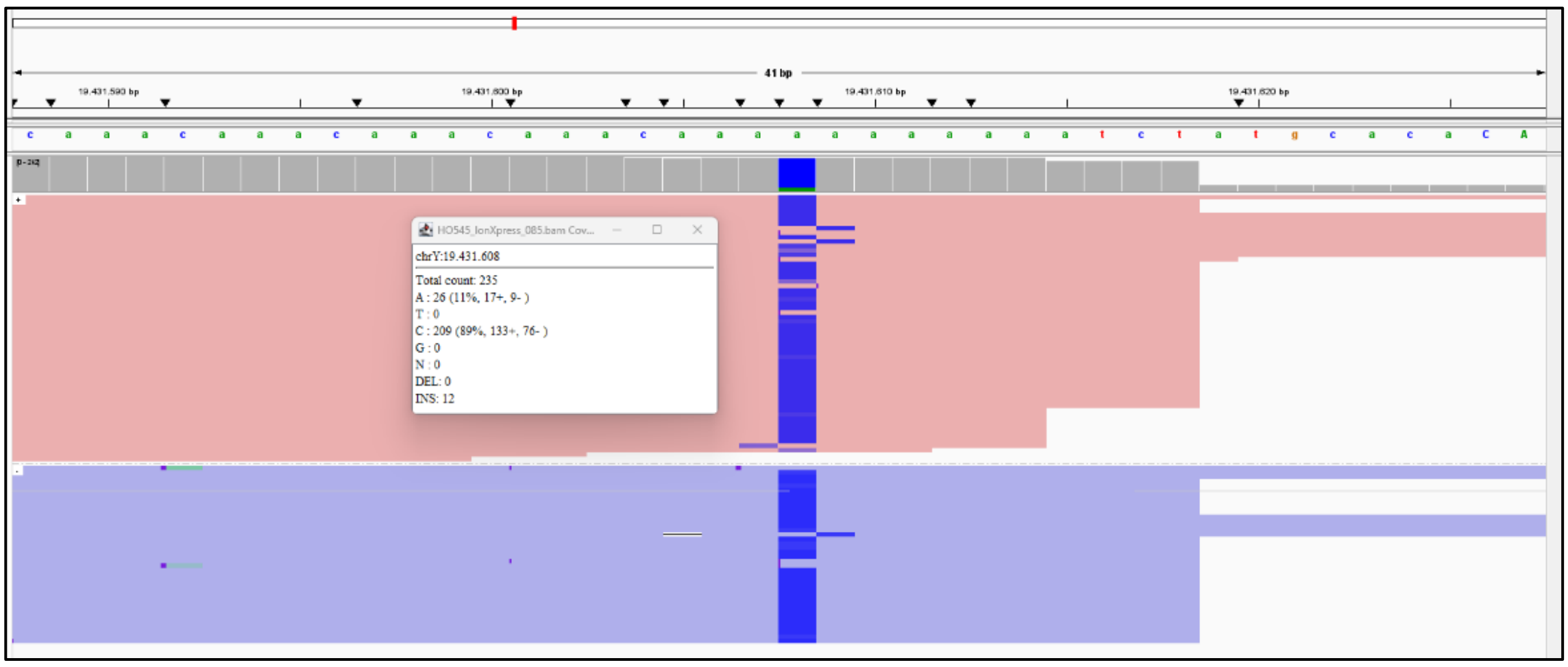

C)

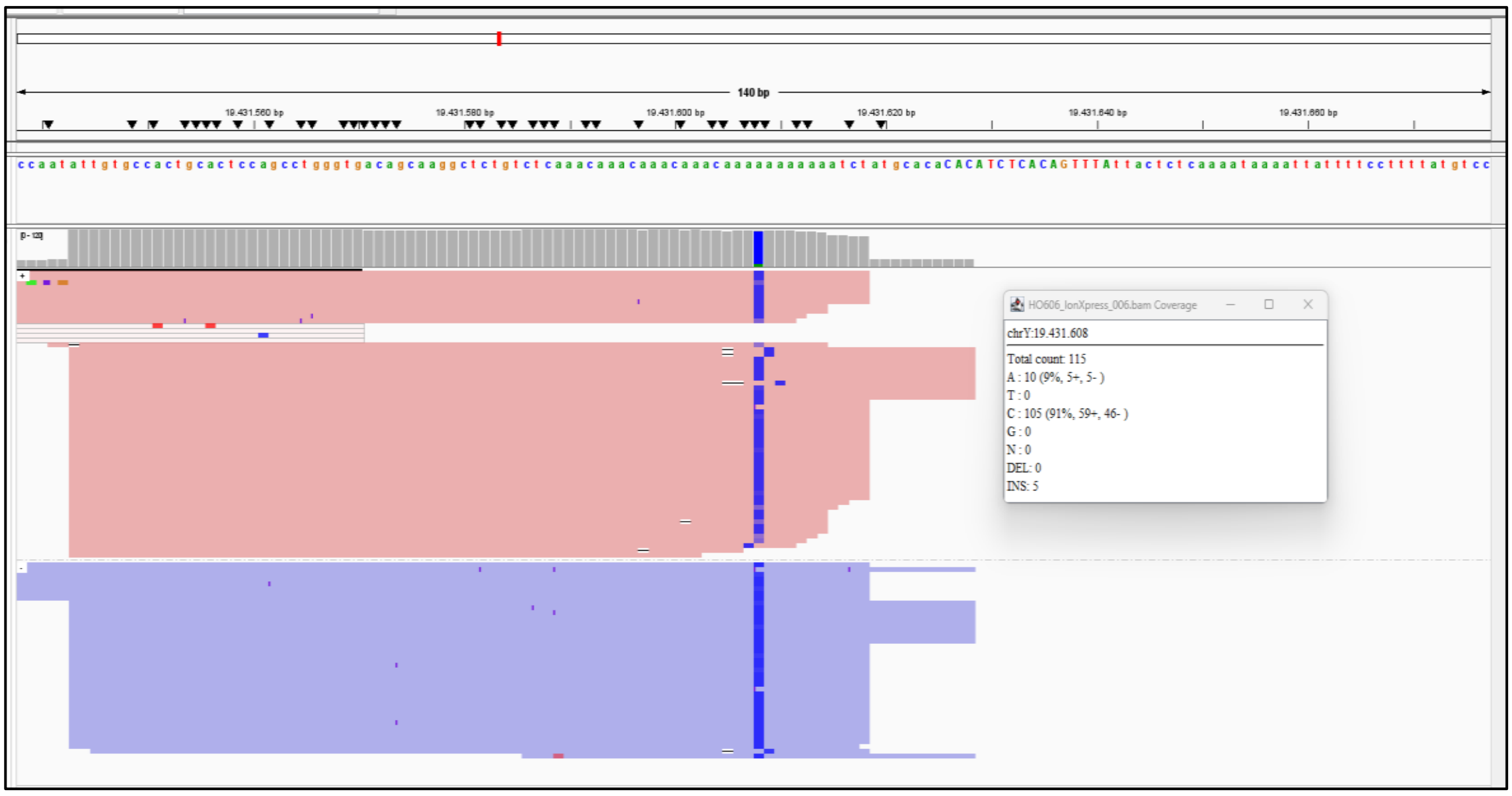

D)

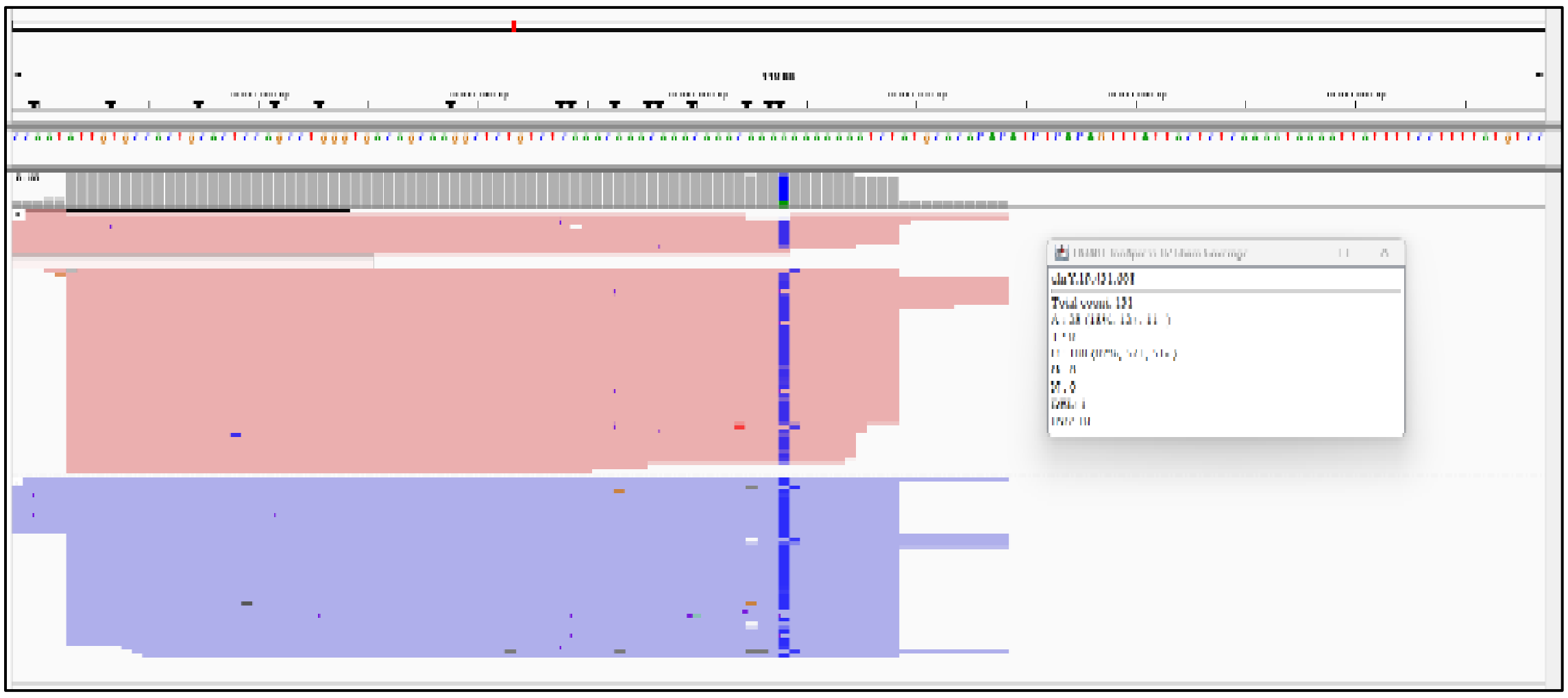

E)

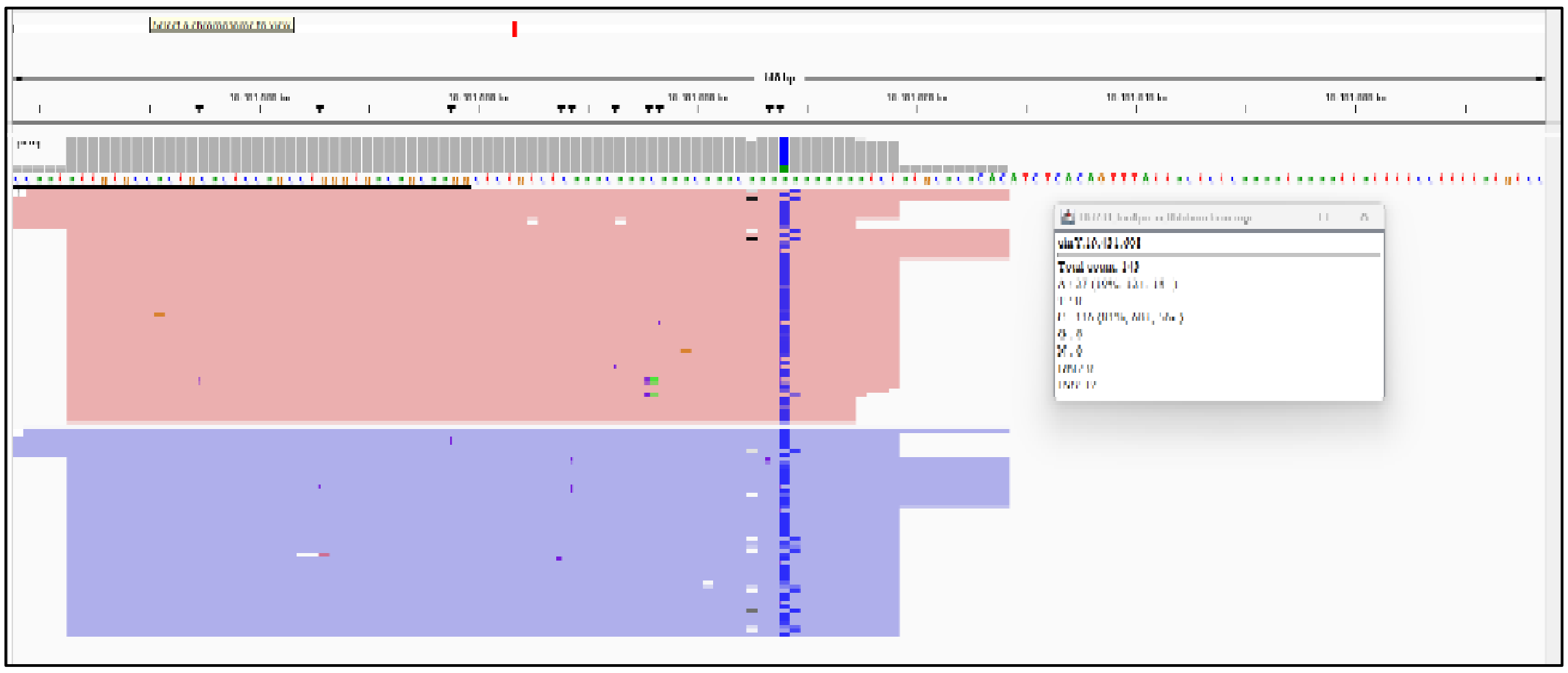

Supplementary Fig. S1. Additional recurrent variation detected in our dataset. A) Andean sample from haplogroup I-M36 with recurrent position M7, usually observed in haplogroup O. B) and C) Andean samples from haplogroup Q-M3 with recurrent position L729, originally reported within haplogroup N. D) Andean sample from haplogroup J-L283 with derived allele for position L729. E) Andean sample from haplogroup R-V88 with derived allele for position L729.

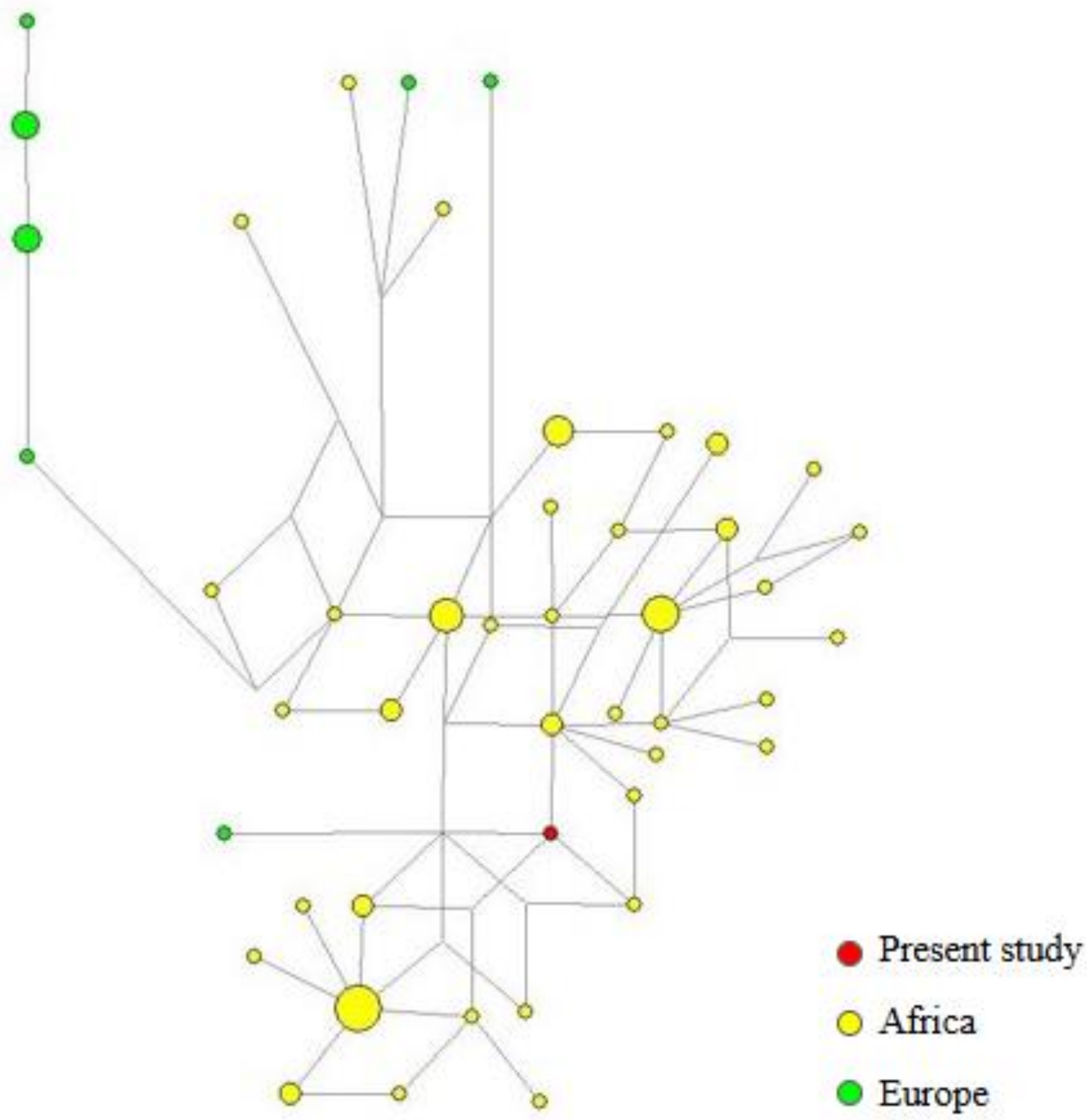

Supplementary Fig. S2. R-V88 median-joining network with 8 Y-STRs (DYS19, DYS385a, DYS385b, DYS389I, DYS389II, DYS390, DYS391 and DYS393) common to all populations. The network includes one sample from this study, 11 from Europe, and 64 from Africa [43-51].
